# Modeling Testosterone Circadian Rhythm in Hypogonadal Males: Effect of Age and Circannual Variations

**DOI:** 10.1208/s12248-015-9841-6

**Published:** 2015-11-09

**Authors:** Mario González-Sales, Olivier Barrière, Pierre-Olivier Tremblay, Fahima Nekka, Julie Desrochers, Mario Tanguay

**Affiliations:** 1 Université de Montréal, Montréal, Canada; 2 inVentiv Health Clinical, 5160 Décarie, Montréal, Canada H3X 2H9

**Keywords:** circadian rhythm, NONMEM^®^, stretched cosine function, testosterone

## Abstract

The objective of this study was to characterize the baseline circadian rhythm of testosterone levels in hypogonadal men. A total of 859 baseline profiles of testosterone from hypogonadal men were included in this analysis. The circadian rhythm of the testosterone was described by a stretched cosine function. Model parameters were estimated using NONMEM^®^ 7.3. The effect of different covariates on the testosterone levels was investigated. Model evaluation was performed using non-parametric bootstrap and predictive checks. A stretched cosine function deeply improved the data goodness of fit compared to the standard trigonometric function (*p* < 0.001; ΔOFV = −204). The effect of the age and the semester, defined as winter and spring *versus* summer and fall, were significantly associated with the baseline levels of testosterone (*p* < 0.001, ΔOFV = −15.6, and *p* < 0.001, ΔOFV = −47.0). Model evaluation procedures such as diagnostic plots, visual predictive check, and non-parametric bootstrap evidenced that the proposed stretched cosine function was able to model the time course of the diurnal testosterone levels in hypogonadal males with accuracy and precision. The circadian rhythm of the testosterone levels was better predicted by the proposed stretched cosine function than a standard cosine function. Testosterone levels decreased by 5.74 ng/dL (2.4%) every 10 years and were 19.3 ng/dL (8.1%) higher during winter and spring compared to summer and fall.

## INTRODUCTION

Testosterone affects almost every organ in the body (1). Functions of testosterone include promoting spermatogenesis, maintenance of accessory organs, muscle growth, development of secondary sexual characteristics, erythropoiesis, bone metabolism, and feedback to the hypothalamus–pituitary (2,3). The normal range for early morning total testosterone in healthy adult males ranges from approximately 300 to 1000 ng/dL (4,5). Low serum levels of total testosterone or hypogonadism (6) have been associated with a number of morbidities including cardiovascular disease, diabetes mellitus, low muscle mass, low bone mass, low physical performance, and frailty (7).

Based on bioequivalence guidelines, the U.S. Food and Drug Administration (FDA) recommends a cutoff value of 250 ng/dL in order to define hypogonadism for clinical trial development and enrollment. In the present analysis, a mean threshold of 300 ng/dL was used to consider a subject as hypogonadal because it was the value used at screening in the available studies, which was in agreement with the common practice. Even though it has been widely discussed, the threshold to consider a subject as hypogonadal is a controversial issue, and different factors have to be considered in the diagnosis of hypogonadism (8). Since a diurnal pattern where the highest level is reached early in the morning is observed (9), hypogonadism diagnosis should be performed within the time period of 8:00–10:00 a.m. (10). Contrary to the substantial evidence for diurnal variation of testosterone, reproducible data demonstrating seasonal patterns of testosterone has been more elusive (11). While some studies suggest its effects (12), others have failed to replicate these results (13). Testosterone concentrations also change as a function of age (14). In healthy men and starting at the third decade of life, there is, on average, a decrease of 1–2% per year (15). Moreover, the prevalence of hypogonadism has recently increased (10). It has been reported that 12%, 19%, 28%, and 49% of men older than 50, 60, 70, or 80 years of age, respectively, suffer from hypogonadism (14). That is the reason why the use of testosterone replacement therapy (TRT) has increased by more than 43% from 2010 to 2013 (16). Historically, the pathway to approval for a TRT required pharmacokinetic but not clinical efficacy criteria (17). Present efficacy endpoints included a requirement that more than 75% of the men treated with TRT reach testosterone levels between the range of 300 and 1000 ng/dL.

The circadian pattern of testosterone has been extensively investigated in healthy males. For example, Gupta *et al.* (18) described the circadian rhythm of testosterone in healthy men; however, they were not able to describe a circadian behavior in hypogonadal men, most likely due to the use of samples after the administration of a transdermal system of testosterone. Moreover, in hypogonadal men, not only are the baseline values lower, but the amplitude of variation is also smaller and thus harder to characterize. Since testosterone is an endogenous substance with a circadian behavior, the assessment of the therapeutic effect of TRT results is challenging. To the best of our knowledge, no model has characterized the time course of the testosterone levels in hypogonadal men under “baseline” or pre-TRT conditions. It should be noted that the term “baseline” is used in the current paper to refer to the absence of TRT. A better understanding of the baseline testosterone kinetics in hypogonadal men would be useful to compare the efficacy between different TRT because the magnitude of the difference in the testosterone levels from the baseline could be considered as the therapeutic TRT effect.

Consequently, the main objective of this analysis was to build a population kinetic model to characterize the circadian rhythm of the baseline testosterone levels in hypogonadal men. This model would be used in the future to compare the efficacy of different TRT. The secondary objectives were to quantify the sources of variability and to explore the impact of patient characteristics on the testosterone kinetic parameters. In this paper, it will be demonstrated that a stretched cosine function is more appropriate to describe the circadian fluctuations in the testosterone levels than a standard cosine function. It will also be shown that the age of the patient and more importantly the time of year have an impact in the baseline levels of testosterone in hypogonadal men.

## MATERIALS AND METHODS

### Study Design and Patient Eligibility Criteria

A total of 859 baseline or pre-dose profiles of testosterone from seven internal studies conducted in hypogonadal men were included in this analysis. Table I provides a summary of the subject demographics by study and overall. Usually, hypogonadism comes with a variety of other complications. Nevertheless, the patients included in these trials had no other medical condition. Subjects were included in these studies if they had mean serum concentrations of testosterone <300 ng/dL and an individual morning serum concentration ≤350 ng/dL, they were 18 years old or older, they had a body mass index (BMI) between 18 and 37 kg/m^2^, and they provided their written informed consent The studies took place in Québec (studies 2–6), Montréal (studies 1–3), Toronto (study 1), North Carolina (studies 3 and 4), Florida (study 4), San Antonio (study 2), and Germany (study 7).

**Table I:** Summary of Hypogonadal Male Characteristics at Baseline Stratified by Clinical Study and Overall

Subject characteristics	Study 1 (*N* = 146)	Study 2 (*N* = 177)	Study 3 (*N* = 131)	Study 4 (*N* = 143)	Study 5 (*N* = 140)	Study 6 (*N* = 85)	Study 7 (*N* = 37)	Overall (*N* = 859)
Age (years)	49.6 (22.0–67.0)	51.3 (22.0–71.0)	50.8 (27.0–72.0)	48.2 (31.0–76.0)	48.0 (21.0–71.0)	51.6 (30.0–72.0)	51.1 (32.0–68.0)	49.9 (21.0–76.0)
Body weight (kg)	86.7 (64.0–110)	87.9 (67.2–117)	86.3 (60.1–131)	86.8 (60.0–116)	88.8 (69.5–117)	82.9 (69.2–105)	90.6 (71.0–117)	87.0 (60.0–131)
Body mass index (kg/m^2^)	28.3 (21.9–32.7)	28.7 (22.6–34.7)	28.7 (21.7–34.8)	28.7 (20.5–35.0)	29.0 (23.4–36.1)	27.8 (24.0–32.9)	28.8 (24.0–34.0)	28.6 (20.5–36.1)
Height (cm)	175 (162–187)	175 (164–194)	173 (164–194)	174 (162–188)	175 (163–186)	172 (162–184)	177 (165–188)	174 (162–194)
Ethnicity (*N*, %)								
Hispanic/Latino	16 (11.0)	44 (24.9)	38 (29.0)	63 (44.1)	26 (18.6)	9 (10.6)	0 (0.0)	196 (22.8)
Non-Hispanic nor Latino	130 (89.0)	133 (75.1)	93 (71.0)	80 (55.9)	114 (81.4)	76 (89.4)	40 (100)	663 (77.2)
Race (*N*, %)								
White	124 (84.9)	161 (91.0)	115 (87.8)	119 (83.2)	140 (100)	82 (96.5)	37 (100)	778 (90.6)
Black	7 (4.8)	16 (9.0)	16 (12.2)	8 (5.6)	0 (0.0)	3 (3.5)	0 (0.0)	50 (5.8)
Asian	7 (4.8)	0 (0.0)	0 (0.0)	3 (2.1)	0 (0.0)	0 (0.0)	0 (0.0)	10 (1.2)
Others	8 (5.5)	0 (0.0)	0 (0.0)	13 (9.1)	0 (0.0)	0 (0.0)	0 (0.0)	21 (2.4)
Season (*N*, %)								
Winter or spring	81 (55.5)	34 (19.2)	131 (100)	0 (0.0)	0 (0.0)	85 (100)	37 (100)	368 (42.8)
Summer or fall	65 (44.5)	143 (80.8)	0 (0.0)	143 (100)	140 (100)	0 (0.0)	0 (0.0)	491 (57.2)

Continuous variables are expressed as median (range), whereas categorical variables are expressed as counts, *N* and percentage (%)

### Blood Sampling Schedule

Testosterone samples from studies 1 to 6 were analyzed by inVentiv Health while testosterone samples in study 7 were analyzed by Clinical Research Services Mannheim GmbH Bioanalytical Laboratory. All the samples were analyzed using a liquid chromatograph with a tandem mass spectrometry detector (LC/MS/MS method). The calibration range of the testosterone assay was 59.22–20,000 pg/mL. The accuracy ranged between 91.10% and 99.99% with a precision ranging between 1.68% and 66.07%. The lower limit of quantification was already provided in the calibration range (59.22 pg/mL). Table II provides a summary of the study characteristics and sampling schedules.

**Table II:** Summary of the Studies’ Characteristics and Sampling Schedules

Sampling times (hours from dose)	Study 1 (*N* = 146)	Study 2 (*N* = 177)	Study 3 (*N* = 131)	Study 4 (*N* = 143)	Study 5 (*N* = 140)	Study 6 (*N* = 85)	Study 7 (*N* = 37)
0	●	●	●	●	●	●	●
−2							●
−4		●	●	●			●
−6					●	●	●
−8	●	●	●	●			●
−10							●
−12		●	●	●	●		●
−14							●
−16	●	●	●	●			●
−18					●	●	●
−20		●	●	●			●
−22							●
−23							●
−24						●	●

● Blood sample was collected at that time

### Pharmacokinetic Model Development

#### Software

Testosterone concentration–time profiles were analyzed using the NONMEM^®^ software (Icon Development Solutions, Ellicott City, MD, USA), which included Version 7.3 installed on a Lenovo ThinkPad T430, equipped with processor Intel i7 3520M, running under Windows 7^®^ 32 bits. Compilations were achieved using gfortran. Model parameters were estimated using stochastic approximation expectation maximization (SAEM) algorithm while standard errors were computed using the Monte Carlo importance sampling (IMP) method. Graphical and all other statistical analyses, including evaluation of NONMEM^®^ outputs, were performed using R version 3.1.0 running under the Rstudio interface.

#### Structural Model

The circadian rhythm of testosterone levels was described using a standard cosine function according to Eq. 1:

1 $$ \mathrm{Circadian}(t)=\mathrm{Amplitude}\cdot cos\left(2\pi \cdot \frac{\left(t-\mathrm{Phase}\right)}{24}\right) $$

where *Amplitude* represents the oscillation in the testosterone levels, *t* represents the time, and *Phase* represents the time at which the peak is achieved.

The complete time course of the testosterone levels was characterized by the addition of a *Base* parameter to the circadian function according to Eq. 2:

2 $$ \mathrm{Testosterone}(t)=\mathrm{Circadian}(t)+\mathrm{Base} $$

where *Base* represents the baseline levels of testosterone.

#### Statistical Model

Visual inspection of individual concentration–time profile suggested interindividual variability. Between-subject or interindividual variability (IIV) in a kinetic parameter, *P*, was included in the model, and it was assumed to be log-normally distributed as shown in Eq. 3:

3 $$ {P}_j={P}^{*}{e}^{\left({\eta}_j\right)} $$

where *P*_*j*_ is the individual kinetic parameter for the *j*th individual, P* is the typical value of the kinetic parameter in the population, and *η*_*j*_ is the parameter quantifying the deviation of the individual parameter from the typical value. *η*_*j*_ was assumed to have zero mean and variance *ω*_*P*_^2^. The magnitude of IIV was expressed as coefficient of variation (CV) calculated as the square root of *ω*_*P*_^2^.

Residual variability was evaluated using a proportional error model according to Eq. 4:

4 $$ {T}_{\mathrm{obs}}={T}_{\mathrm{pred}}\cdot \left(1+\varepsilon \right) $$

where *T*_obs_ is the observed testosterone level, *T*_pred_ is the corresponding model prediction, and *ε* is a parameter quantifying the residual variability, assumed to follow an independent Gaussian distribution with mean zero and variance *σ* ^2^.

#### Model Selection Criteria

To identify the best statistical model, a series of models were evaluated. For each model, the improvement in the fit obtained was assessed by the likelihood ratio test (LRT) (*p* = 0.001), the reduction in the IIV and residual variability, the precision in parameter estimates, the examination of diagnostic plots, and the shrinkage (19).

#### Covariate Analysis

The effect of age, body weight, body mass index (BMI), height, ethnicity, season, and semester was explored as sources of IIV on the testosterone kinetic parameters. In order to investigate a possible seasonal effect, season was defined as a covariate with four categories: winter, spring, summer, and fall. Winter, spring, summer, and fall were considered from December 21st to March 19th, from March 20th to June 19th, from June 20th to September 21st, and from September 22nd to December 20th, respectively. Semester was split into two subcovariates. On the one hand, semester 1 was defined as a dichotomous variable with a value of 0, if summer or fall, and a value of 1, if winter or spring. On the other hand, semester 2 was defined as a dichotomous variable with a value of 0, if spring or summer, and a value of 1, if fall or winter. Similarly, ethnicity was defined as a value of 0 for Hispanic or Latino and a value of 1 for non-Hispanic nor Latino. Race was not evaluated because its categories were unbalanced. A screening analysis was conducted only on the kinetic parameters where the shrinkage was lower than 0.3 and was based on visual graphical inspection and stepwise linear regression of the relationships between the individual Bayesian kinetic parameters and the covariates. Only covariates with statistically significant (*p* < 0.05) and potentially clinically relevant (*r* ^2^ > 0.2) effect on kinetic parameters in the screening analysis were further tested one by one in NONMEM^®^ in order to be incorporated in the population model.

The effect of selected covariates on model parameters was explored following the forward-inclusion (*p* < 0.005) and backward-elimination (*p* < 0.001) process as described elsewhere (20). With this methodology, only covariates showing significant contributions were kept in the population model. Categorical covariates were incorporated into the model as index variables, whereas continuous covariates were included using linear equations after centering on the median.

#### Model Evaluation

A visual predictive check was made using the technique described by Yano *et al.* (21). The parameter estimates obtained by fitting the selected population kinetic model to the data were used to simulate the population kinetic profile of the testosterone levels. Using model predictions, a non-parametric 90% prediction interval around the observed testosterone levels was constructed to quantify the variability in the model predictions. Furthermore, normalized prediction distribution error (NPDE) was also assessed (22). If the model adequately describes the data, NPDE should follow a normal distribution with mean 0 and variance 1. Finally, a non-parametric bootstrap was used as internal evaluation method to qualify the estimates of the model parameters (23). The mean and the 95% confidence intervals (CI) of the parameter estimates from the bootstrap replicates were compared with the estimated parameters from the original dataset.

## RESULTS

The dataset analyzed in this study included seven internal studies conducted at inVentiv Health. The model fitted a total of 859 baseline profiles of testosterone consisting of 4556 observations. Table I summarizes the characteristics of the patients included in this study. The internal model evaluations were conducted under the same dataset. Model parameters were estimated using SAEM algorithm followed by IMP method in order to compute the standard error and to obtain a value of the objective function to compare between models.

**Fig. 1:**
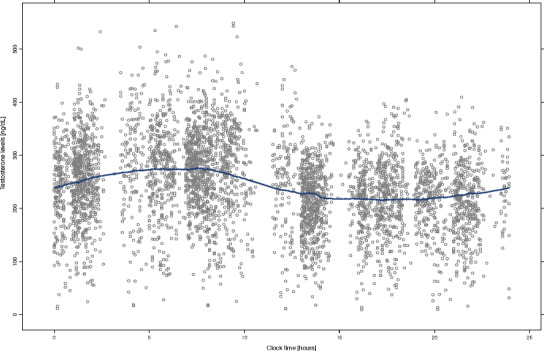
Time course of the baseline testosterone levels in hypogonadal men. *Gray points* represent observations. The *blue line* represents the smooth regression line

A standard cosine function provided a reasonable description of the time course of the baseline testosterone levels. However, the plot of the testosterone levels *versus* time (Fig. 1) revealed that after the morning peak, the decrease in the testosterone levels is faster than the increase from the nadir. This fact suggests an asymmetric circadian rhythm of testosterone. Consequently, a stretched cosine function was proposed. This function allows the time between the peak of testosterone (*t*_max_) and the nadir (*t*_min_) to be different from 12 h in order to capture this non-symmetric behavior. This equation has several components according to Eqs. (5–7):

5 $$ {t}_{24}=\left(t-{t}_{\max}\right) \mod\;24 $$

where *t*_24_ is a vector of the difference between time *t* and *t*_max_ *modulo* 24 h, that is, limited to a 24-h interval.

6 $$ {L}_1=\left({t}_{\min }-{t}_{\max}\right) \mod\;24 $$

where *L*_1_ is the length of the first interval, in other words the time that it takes from the peak of testosterone to the nadir *modulo* 24 h as well.

7 $$ {L}_2=24-{L}_1 $$

*L*_2_ represents the length of the second interval, and thus, the stretch of the cosine function (*τ*) can be defined as shown in Eq. 8:

8 $$ \tau \left\{\begin{array}{l}\mathrm{if}\;{t}_{24}\le {L}_1\;\mathrm{then}\;\tau =\frac{12}{L_1}{t}_{24}\hfill \\ {}\mathrm{if}\;{t}_{24}>{L}_1\;\mathrm{then}\;\tau =12+\frac{12}{L_2}\left({t}_{24}-{L}_1\right)\hfill \end{array}\right. $$

The new parameters to be estimated were *t*_max_ and *t*_min_ instead of the phase shift, and as can be observed, *τ* depends on *t*_max_, *t*_min_, and *t*. Therefore, the asymmetric circadian rhythm can be defined according to Eq. 9:

9 $$ \mathrm{Circadian}(t)=\mathrm{Amplitude}\cdot \cos \left(2\pi \cdot \frac{\tau }{24}\right) $$

This parameterization had a major impact in the objective function value (OFV), which decreases by 84 points compared to the standard trigonometric function. Also, compared to a double cosine function, the decrease was 40 points.

Moreover, the goodness of fit was further improved by the inclusion of IIV in *t*_max_ (∆OFV = −49.9), *t*_min_ (∆OFV = −196), *Amplitude* (∆OFV = −125), and *Base* (∆OFV = −3980). However, etabar problems in *Base* were noticed. In this sense, the histograms and density plots of the distribution of the eta values in *Base* brought to light a certain degree of skewness. Consequently, a Box–Cox transformation in *η*_Base_ was implemented as described in Eq. 10:

10 $$ {\eta}_{\mathrm{Base}}=\frac{\left({\left({e}^{\eta_{\mathrm{Base}}}\right)}^{\theta_1}-1\right)}{\theta_1} $$

where *θ*_1_ is a parameter to be estimated characterizing the skewness of the distribution. Under this codification, the etabar problems disappeared and a significant reduction in the OFV was achieved (∆OFV = −107). Moreover, at the first stage of the modeling, independence between the interindividual random effects was assumed. However, the graphical and statistical explorations of the correlations among the IIV terms revealed a potential association between *t*_max_ and *t*_min_ and between *Amplitude* and *Base*. The correlations between these parameters were further evaluated in NONMEM^®^ by estimating the non-diagonal elements of the variance–covariance matrix for the between-subject random effects. The estimation of the off-diagonal elements of the omega correlation matrix further improved the model fit to the data (∆OFV = −150). For this model, the shrinkage of the interindividual random effects of *t*_max_, *t*_min_, *Amplitude*, and *Base* was 61%, 47%, 29%, and 4%, respectively.

**Fig. 2:**
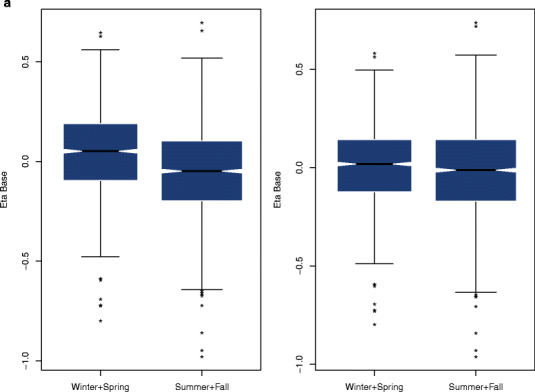

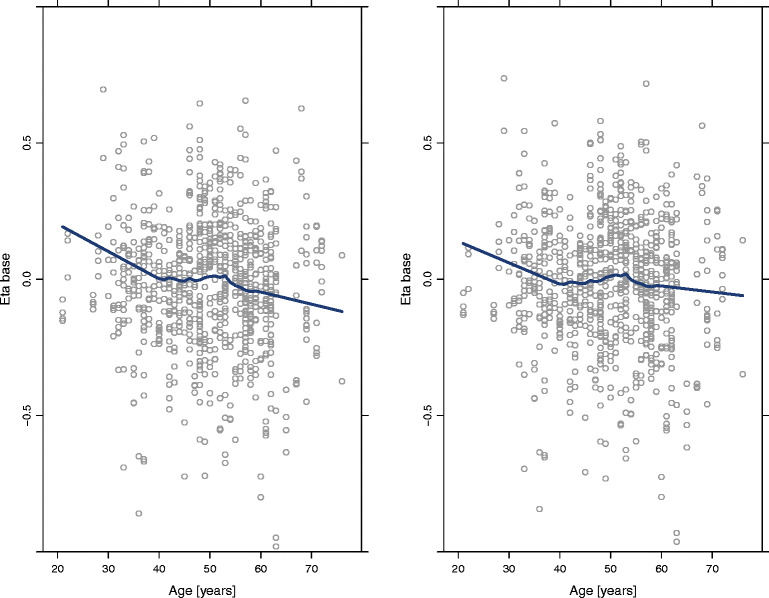
**a** Distribution of the interindividual variability (eta) of *Base* by season before (*left panel*) and after (*right panel*) including the effect of season in the model. *Asterisks* represent outliers. **b** Correlation between the interindividual variability (eta) of *Base* and age before (*left panel*) and after (*right panel*) including the effect of age in the model. The *blue solid line* represents the smooth regression line

The next step in the model building process was the covariate analysis. Height was not tested because it was included in the computation of the BMI. Out of 859 subjects enrolled in this analysis, 778 (90.6%) were white or Caucasian. Consequently, race was not evaluated because its categories were not balanced. Scatterplots (for continuous) or boxplots (for categorical) of the interindividual random effects *versus* covariates were created in those parameters where the shrinkage was lower than 0.3 (*Amplitude* and *Base*). This graphical analysis reveals a slight trend between the baseline and the age (Fig. 2a) and between the baseline and the semester (winter and spring *vs* summer and fall) (Fig. 2b). The inclusion of the effect of age and semester on *Base* improved the model fit to the data and decreased the OFV in a significant extent (∆OFV = −15.6 and ∆OFV = −47.0, respectively). Age and semester explained 2.5% and 3.4% of the variability in the baseline values of testosterone. The search for sources of IIV in those parameters where the shrinkage was higher than 0.3 (*t*_max_ and *t*_min_) was conducted with a formal analysis in NONMEM^®^ after exploring all the combinations. No additional covariates other than the age and the season were found to explain IIV in any parameter nor improved the model fit to the data. Consequently, the model described was considered the best model to describe the time course of the baseline levels in hypogonadal men.

**Fig. 3:**
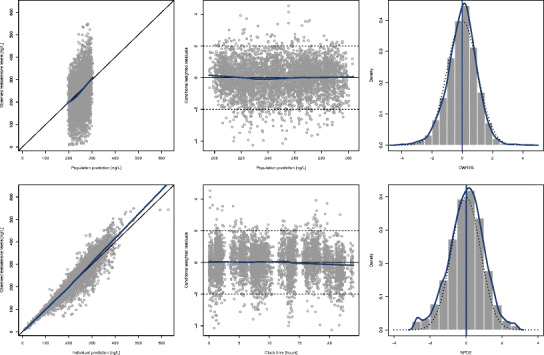
Diagnostic plots of the model developed to characterize testosterone kinetics in hypogonadal men. *Left panels* represent the scatterplots of observations and population predictions (*upper panel*, **a**) and the observations and individual predictions (*lower panel*, **b**). *Middle panels* show scatterplots of conditional weighted residuals and population predictions (*upper panel*, **c**) and time (*lower panel*, **d**). *Right panels* represent the density plots of conditional weighted residuals (*upper panel*, **e**) and normalized prediction distribution errors (*lower panel*, **f**). *Blue lines* represent the smooth regression line in scatterplots and the density line in density plots. *Dotted black lines* represent the theoretical density line

Plots of observations *versus* population (Fig. 3a) and individual (Fig. 3b) predicted concentration, and conditional weighted residuals (CWRES) *versus* observations (Fig. 3c) and *versus* time (Fig. 3d) showed random uniform scatter around the identity line, indicating the absence of bias while histograms of CWRES (Fig. 3e) and NPDE (Fig. 3f) exhibited centered distribution around 0. The parameter estimates and their associated precisions, measured as relative standard error (RSE), for the best model are presented in Table III. All structural model parameters, or fixed effects, were estimated with good precision (RSE < 33%), and random effects were estimated with very good precision (RSE < 17%).

**Table III:** Non-parametric Bootstrap Analysis and Parameter Estimates (Relative Standard Errors) of the Testosterone Population Kinetic Model

Model parameters	Original dataset	Non-parametric Bootstrap (*N* = 1000 replicates)	
	Estimate (RSE%)	Mean	95% confidence interval
*Base* (ng/dL)	239 (1.1)	239	232, 245
*Amplitude* (ng/dL)	32.1 (3.5)	32.2	30.2, 34.1
*t*_max_ (clock time)	9:22 (0.4)	9:21	8:13, 10:30
*t*_min_ (clock time)	14:02 (0.4)	14:00	12:49, 15:33
Season on *Base* (%)	8.09 (18.8)	7.86	4.66, 11.0
Age on *Base* (%/10 years)	−2.40 (24.0)	−2.08	−0.07, −3.55
Box–Cox on *Base*	−1.93 (2.4)	−1.98	−1.69, −2.30
Interindividual variability (CV%)			
*η*_*Base*_	25.1 (3.6)	25.1	23.1, 27
*η*_*Amplitude*_	50.3 (6.6)	50.2	43.5, 56.0
${\eta}_{t_{\max }}$	10.6 (19.1)	10.4	7.7, 14.4
${\eta}_{t_{\min }}$	19.0 (11.5)	19.4	16.0, 22.1
Residual variability (CV%)			
*σ*_proportional_	13.8 (2.4)	13.8	13.1, 14.4

Shrinkage values for *t*_max_, *t*_min_, *Amplitude*, *Base* parameters, and residual variability were 60, 46, 27, 4, and 2%, respectively

*Base* baseline value, *t*_*max*_ time at which the peak of testosterone is reached, *t*_*min*_ time at which the nadir of testosterone occurs

**Fig. 4:**
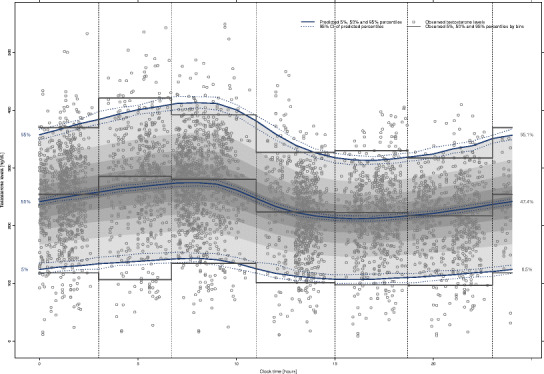
Visual predictive check of the model developed to characterize the time course of the testosterone levels in hypogonadal men. *Blue lines* represent the predicted 5th, 50th, and 95th percentiles of the testosterone concentrations. *Dotted blue lines* represent the 95% prediction interval (PI) of these percentiles. The *shaded gray area* represents the 90% prediction interval (PI) predicted for the model. *Vertical dotted lines* represent the selected bins. *Gray solid lines* represent the observed 5th, 50th, and 95th percentiles of testosterone concentrations in each bin. *Figures* at the *margin* of the plot represent the theoretical (*left*) and observed (*right*) percentage of observations below percentiles 5th, 50th, and 95th

The results of the visual predictive check performed are presented in Fig. 4. In this plot, the blue lines represent the percentiles 5th, 50th, and 95th based on the model predictions and the shaded gray area between them represents the 90% prediction interval (PI). For each of these percentiles, a 95% PI was constructed (dotted blue lines around these percentiles). The vertical dotted lines represent the selected bins. For each bin, the percentiles 5th, 50th, and 95th based on the observations were computed and plotted on top of model prediction (horizontal blue solid lines), in order to visually compare the variability in the model predictions. Besides, the percentage of observations below the percentiles 5th, 50th, and 95th was computed and plotted on the right margin of the plot. In this sense, 6.5%, 47.4%, and 95.1% of the observations were below the theoretical 5%, 50%, and 95%. This figure evidences that the population kinetic model developed was deeply appropriate to characterize the time course of the baseline testosterone levels in hypogonadal men. Furthermore, the mean of NPDE was 0.019 (95% CI −0.011, 0.044) and the value of the standard deviation was 0.986 (95% CI 0.964, 1.008).

Non-parametric bootstrap analysis was also used as an internal model evaluation technique to qualify the developed model. From the 1000 bootstrap replicates, 0 (0%) failed to minimize successfully. The analysis of the results from the 1000 bootstrap replicates that minimized successfully is provided in Table III. The population estimates for the final model were very similar to the mean of the 1000 bootstrap replicates, and were contained within the 95% confidence intervals obtained from the bootstrap analysis. Besides the accuracy, the precision of the NONMEM^®^ parameter estimates was also good, because the relative standard error from the bootstrap analysis for the fixed and random effects were lower than 14% and 3%, respectively. Overall, the validation results are satisfactory for this dataset.

## DISCUSSION

The primary goal of this analysis was to build a population kinetic model in order to characterize the circadian rhythm of the baseline testosterone levels in hypogonadal men. A stretched cosine function was used to describe the non-symmetric behavior in the circadian rhythm of the testosterone levels. According to the estimated model parameters, the time that it takes from the peak of testosterone to the nadir was estimated to be around 5 h. This means that after reaching the nadir, the increase until the new peak is much slower (approximately 19 h). In this sense, it was estimated that the peak of testosterone occurs around 9:20 in the morning, which is consistent with the clinical practice for the diagnosis of hypogonadism (10). In this study, a cutoff of 300 ng/dL was used to consider a subject with low levels of testosterone. Accordingly, the base value of testosterone in hypogonadal men was estimated to be 239 ng/dL. Moreover, the amplitude of the oscillation was estimated to be 32.4 ng/dL, with an associated IIV higher than 50%. Therefore, the modeled typical hypogonadal patient would always be considered as a hypogonadal subject, even at the peak testosterone concentration.

During the model building process, a Box–Cox transformation was implemented because etabar problems in the *Base* parameter were noticed. In population modeling, it is commonly assumed that eta parameters follow a normal distribution with a mean of 0 and a variance of *ω* ^2^. However, this is not always true as reported by Petterson *et al.* (24). The authors’ suggested some transformation to relax the often erroneous assumption of a known shape of the parameter distribution. In our model, the additional parameter estimated required for the Box–Cox transformation was close to a value of −2, indicating negative skewness.

Within the range of values analyzed, body weight, ethnicity, and smoke did not influence the circadian rhythm of testosterone to a significant extent. However, an increase in age was associated with a decrement in the baseline values of testosterone. Concretely, it was estimated that during a 10-year span, the baseline concentration of testosterone would be 5.74 ng/dL lower at the end of the period compared to the beginning. These results are in concordance with the known decrease in the testosterone levels with age (15). More interesting was the fact that a seasonal effect was demonstrated. Baseline testosterone levels were 8.09% higher during the winter and spring compared to the summer and fall. This difference might be clinically relevant because, on average, a difference of 19.3 ng/dL is expected between seasons. These results are in accordance with the findings of Svartberg *et al.* (12). Effectively, in a study conducted in Norway, these authors observed that free testosterone levels showed a significant seasonal pattern (*P* < 0.001), with the peak in December and the nadir in August. Lowest testosterone levels occurred in months with the highest temperatures and longest hours of daylight. In our study, one explanation of the seasonal effect might be the variations in temperature and daylight in the provinces of Québec and Ontario, Canada, where the studies mainly took place. The average temperatures in this area during winter and spring are much lower than during the summer and fall, and also, on average, the days are around 150 min shorter in terms of sunlight. In this sense, it has been observed that lower temperatures stimulate the pituitary gland to release luteinizing hormone (LH) and follicle-stimulating hormone (FSH) during “cold” seasons (25). Besides, the peak of LH and FSH occurs in March and February, respectively. It is also known that physiologically, FSH and LH stimulate the testes to synthesize testosterone and, consequently, the authors’ hypothesis that the seasonal effect on the baseline values of testosterone was due to an indirect effect because of the low temperatures and the shorter hours of daylight. However, this effect might not be observed in other parts of the world where the temperature variations are not as important as in the northern countries.

**Fig. 5:**
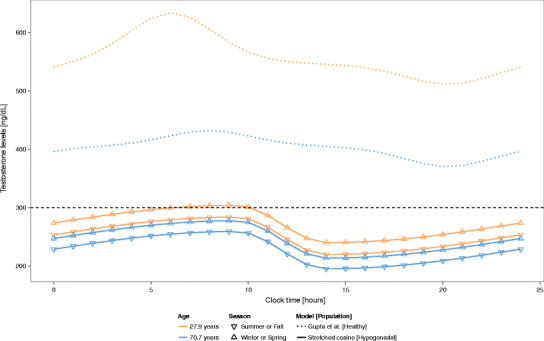
Simulation of the time course of the testosterone levels based on typical patient values. The *horizontal black dashed line* represents the threshold for hypogonadism (300 ng/dL)

Model-based simulations of the time course of the testosterone levels for young (27.9 years old) and old (70.7 years old) subjects during winter and spring and summer and fall are presented in Fig. 5. These simulations are presented together with the time course of testosterone levels in the typical young and old healthy subjects based on the model developed by Gupta *et al.* (18). Both models predict similar patterns with higher testosterone peaks early during the morning and nadir levels during the afternoon and evening. As expected, the simulations evidenced that lower levels of testosterone would be easier observed in an old subject compared to a young subject. Moreover, one subject would be more likely diagnosed as hypogonadal during summer and fall compared to winter and spring. Furthermore, it should be noted that the testosterone levels of a young hypogonadal male during summer and fall will be similar to the levels of an old hypogonadal male during winter and spring. The simulations also evidence that a fast decrease in the testosterone levels occurs after the testosterone peak. This fact might show the clinicians the optimal time for the TRT to be administrated in order to maintain the testosterone levels within the normal range of 300 and 1000 ng/dL.

Visual predictive check, normalized predictive distribution errors, and non-parametric bootstrap were used as complementary tools to internally evaluate the model developed. The bootstrap analyses conducted in the current study yielded mean model parameters that were comparable to the estimates of the original data set, indicating the stability of the developed model. These findings were further supported by the predictive checks performed, which confirmed the model developed was suitable to describe the time course of testosterone levels in hypogonadal men. As a result, the efficacy between different TRT can be assessed by the proposed model described above. Effectively, the time course of the testosterone levels in hypogonadal men depending on the age and the season of the year can be simulated from our model. These profiles can be compared with the profiles after the administration of TRT, and thus, the therapeutic effect of different TRT would be easily evaluated. Moreover, because of the use of a stretched cosine function, it can be known that the lowest values of testosterone after the peak occur faster than a 12-h interval. Therefore, a more accurate dose regimen can be proposed to minimize the impact of the circadian behavior of testosterone in hypogonadal men.

In summary, we demonstrated that a stretched cosine function where the circadian rhythm was considered to be asymmetric was the most suitable function to describe the circadian behavior of the baseline testosterone levels in hypogonadal men. The proposed stretched cosine function might also be useful in all those situations where a non-symmetric circadian rhythm is observed in order to improve model predictions. Age and season were identified as predictors of the baseline testosterone levels. The present model manages to well characterize the population and individual kinetics of the testosterone levels, making it suitable to compare the efficacy between different TRT.
